# Variable Freshwater Influences on the Abundance of *Vibrio vulnificus* in a Tropical Urban Estuary

**DOI:** 10.1128/aem.01884-21

**Published:** 2022-03-22

**Authors:** Olivia D. Nigro, La’Toya I. James-Davis, Eric Heinen De Carlo, Yuan-Hui Li, Grieg F. Steward

**Affiliations:** a Department of Natural Science, Hawaiʻi Pacific University, Honolulu, Hawaiʻi, USA; b Daniel K. Inouye Center for Microbial Oceanography—Research and Education, School of Ocean and Earth Science and Technology (SOEST), University of Hawaiʻi at Mānoa, Honolulu, Hawaiʻi, USA; c Department of Oceanography, School of Ocean and Earth Science and Technology (SOEST), University of Hawaiʻi at Mānoa, Honolulu, Hawaiʻi, USA; Norwegian University of Life Sciences

**Keywords:** *Vibrio vulnificus*, genotypic identification, seasonal cycle, tropical estuary

## Abstract

To better understand the controls on the opportunistic human pathogen Vibrio vulnificus in warm tropical waters, we conducted a year-long investigation in the Ala Wai Canal, a channelized estuary in Honolulu, HI. The abundance of V. vulnificus, as determined by quantitative PCR (qPCR) of the hemolysin gene (*vvhA*), varied spatially and temporally by nearly 4 orders of magnitude (≤3 to 14,000 mL^−1^). Unlike in temperate and subtropical systems, temperatures were persistently warm (19 to 31°C) and explained little of the variability in V. vulnificus abundance. Salinity (1 to 36 ppt) had a significant, but nonlinear, relationship with V. vulnificus abundance with the highest *vvhA* concentrations (>2,500 mL^−1^) observed only at salinities from 7 to 22 ppt. V. vulnificus abundances were lower on average during the summer dry season, when waters were warmer but more saline. The highest canal-wide average abundances were observed during a time of modest rainfall, when moderate salinities and elevated concentrations of reduced nitrogen species and silica suggested a groundwater influence. Parallel quantification of the *vcgC* gene suggested that C-type strains, which are responsible for most human infections, comprised 25% of the total V. vulnificus on average, but their relative contribution was greater at higher salinities, suggesting a broader salinity tolerance. Generalized regression models suggested that up to 67% of sample-to-sample variation (*n* = 202) in log-transformed V. vulnificus abundance was explained using the measured environmental variables, and up to 97% of the monthly variation in canal-wide average concentrations (*n* = 13) was explained with the best subset of four variables.

**IMPORTANCE** Our data illustrate that, in the absence of strong seasonal variation in water temperature in the tropics, variation in salinity driven by rainfall becomes a primary controlling variable on V. vulnificus abundance. There is thus a tendency for a rainfall-driven seasonal cycle in V. vulnificus abundance which is inverted from the temperature-driven seasonal cycle at higher latitudes. However, stochasticity in rainfall and its nonlinear, indirect effects on V. vulnificus concentration means that high abundances can occur at any location in the canal at any time of year, making it challenging to predict concentrations of this pathogen at a high temporal or spatial resolution. Much of the variability in canal-wide average concentrations, on the other hand, was explained by a few variables that reflect the magnitude of freshwater input to the system, suggesting that relative risk of exposure to this pathogen could be predicted as an average for the system.

## INTRODUCTION

The bacterium V. vulnificus is an opportunistic and formidable human pathogen that has a worldwide distribution in a variety of marine and estuarine environments (1, 2). In humans, V. vulnificus may cause a range of illnesses including gastroenteritis, necrotizing fasciitis, and septicemia (3). Infections occur as a result of ingestion of contaminated seafood (4) or via wound exposure to water (5). Strains vary in their propensity to cause disease in humans, with certain genotypically distinguishable strains much more commonly, but not exclusively, associated with disease in humans (6). The exact mechanisms of virulence in V. vulnificus, and the genes responsible for the onset of illness, have yet to be determined, but a number of correlative biomarkers have been used to discriminate those strains most commonly associated with human disease (7). Variations in the 16S rRNA gene, for example, have been used in PCR assays to discriminate “A-type” strains from “B-type” strains (8, 9), the latter of which predominate among clinical isolates. Another commonly used marker is the 200-bp segment of the virulence-correlated gene that resolves the gene variants *vcgC* or “C-type” strains from *vcgE* or “E-type” strains (10). PCR-based analysis of 55 V. vulnificus isolates indicated that 90% of the strains isolated from infected patients were C-type (clinical), while 93% of the strains isolated from environmental samples were E-type (environmental). Subsequent analyses revealed broader genomic differences along with physiological differences between these lineages, suggesting that they are distinct ecotypes that may be better adapted for either environmental growth (E-type) or stress tolerance (C-type) (11). These biomarkers are largely congruent, with the common environmental strains being A-type/E-type and the majority of clinical isolates being B-type/C-type, although either type can cause disease in humans (12).

Studies of V. vulnificus in temperate and subtropical waters have shown that warmer temperatures increase the frequency of detection (13–16). Quantification over an annual cycle reveals a clear temperature-driven seasonal signal, with the highest concentrations of V. vulnificus occurring during warm summer months (17–20) and culturable cells declining dramatically at temperatures below 13°C (7). V. vulnificus abundance is also influenced by salinity (20–23), thriving in conditions of both warm temperatures and moderate salinities (6). The environmental abundance patterns are consistent with observations of V. vulnificus growth under controlled laboratory conditions (13, 24), which showed increasing growth rates up to around 37°C, and a broad salinity tolerance with highest growth rates between 5 and 25 ppt. Correlation models of environmental data support the idea that temperature and salinity are two of the most important variables controlling V. vulnificus abundance, but their relative importance depends on the ranges over which they are sampled (22, 25–28).

In temperate environments, the incidence of V. vulnificus infection tracks the seasonal environmental abundances of the pathogen, with the most infections occurring during the warm summer months (7, 29). It follows that the inhabitants of subtropical and especially tropical areas, where air and water temperatures are warm year-round, would be particularly vulnerable to V. vulnificus infection. Indeed, according to available surveillance data for the years 2003 to 2008 (30–32), Hawaii had the fifth highest incidence of non-foodborne V. vulnificus infections in the US, trailing only four gulf states (FL, LA, MI, and TX). When we convert these rates to a per capita basis, the rate for Hawaii was the highest in the nation. Despite the higher incidence of V. vulnificus wound infections, primarily from recreational waters, there has been little data collected on V. vulnificus in the coastal waters of Hawaiʻi (33, 34) and scant data on the ecology of V. vulnificus in tropical waters in general (21). Consequently, we initiated an investigation of the abundance and dynamics of V. vulnificus in the Ala Wai Canal and Harbor.

The Ala Wai Canal provides partially channelized drainage for two watersheds. Although it is not designated as a recreational waterway, the canal is used extensively for boating and fishing. Flow down the canal varies as a function of tide and rainfall, the latter driving surface runoff (streams and storm drains) and, with some hysteresis, groundwater seepage. Salinity varies widely in the canal as a function of depth, overall stream flow, position in the canal relative to freshwater sources, and tidal forcing. Water temperature, on the other hand, varies over a relatively narrow range compared to that in temperate systems. Because of the seasonality of rainfall in Hawaii, with higher precipitation during winter months (35), we hypothesized that there could be an inverse seasonal pattern in V. vulnificus abundance that is driven by salinity, in contrast to the strongly temperature-driven patterns in temperate waters.

Our objectives with this study were to document the temporal and spatial variability of V. vulnificus total abundance and the proportion that is C-type in the estuarine waters of the Ala Wai Canal and Harbor, and to determine how this abundance was related to environmental variables. The goal was to better understand the environmental controls on V. vulnificus in tropical estuarine waters and to assess the prospects for modeling pathogen abundance.

## RESULTS

### Variability of the habitat.

**FIG 1:**
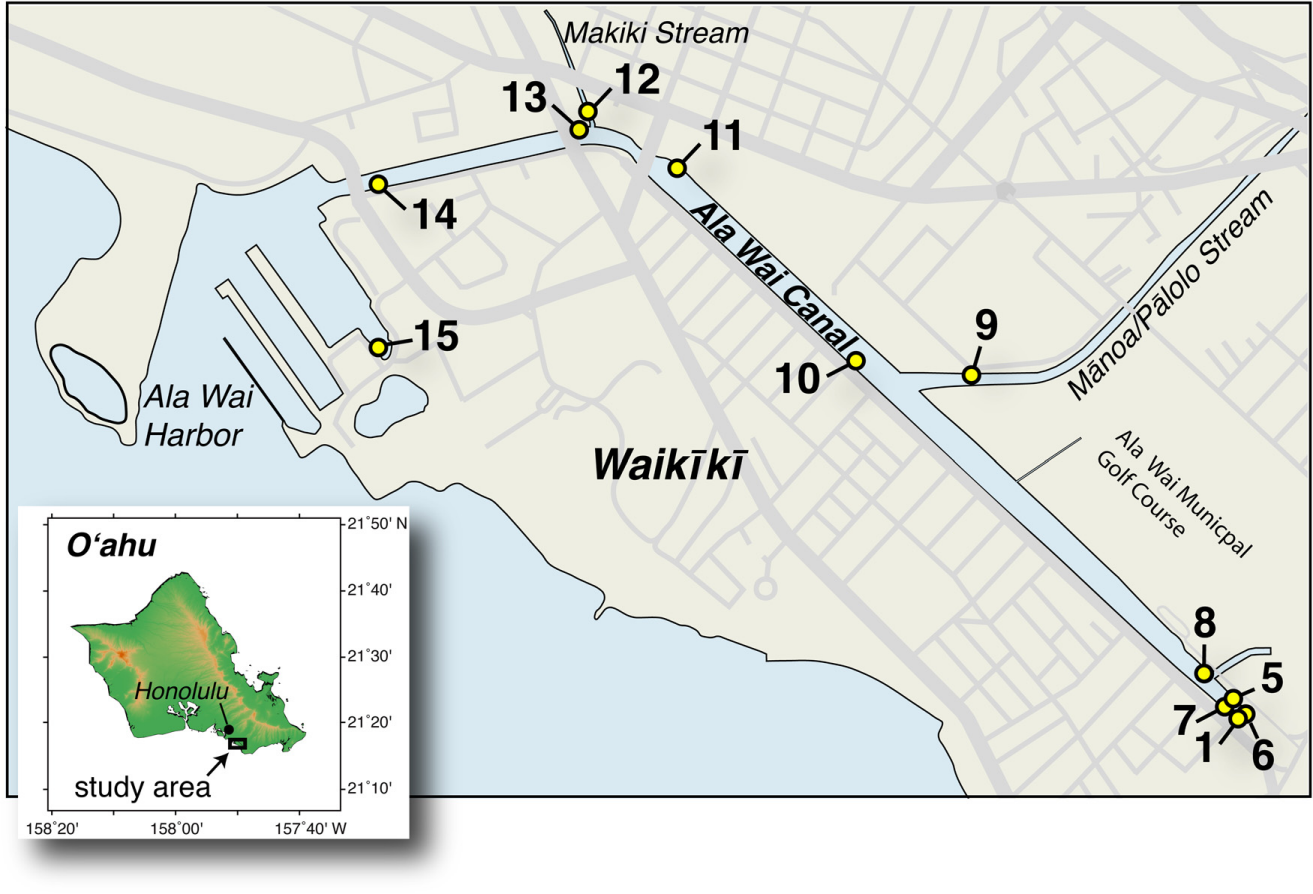
Map of sampling sites. Inset shows the general location of the canal on the south shore of the island of Oʻahu in the Hawaiian Island chain. Main map shows site numbers and position along the canal. Site 1 is at the closed end of the canal with occasional input from surface runoff via storm drains. Sites 9 and 12 are at the mouths of the Mānoa-Pālolo and Makiki streams, respectively. Main map of sampling site locations was drawn and shaded by hand in Adobe Illustrator using a United States Geological Survey (USGS) base map (https://dashboard.waterdata.usgs.gov) as a template for the coastline and roads, and inset map of O’ahu was produced using Generic Mapping Tools 5.2.1 (https://www.generic-mapping-tools.org/).

Over the course of a 13-month study at 12 sites in the Ala Wai Canal and Harbor (Fig. 1; Table S1 in the supplemental material) we measured a suite of *in situ* physical, chemical, and biological properties of the surface water, along with environmental variables that influence the freshwater balance of the canal. Rainfall in Mānoa Valley, one of the major watersheds draining into the canal, varied from 0 to 15.8 cm over the 24-h period preceding each sampling. The average rainfall prior to samplings in the rainy season (October to March) was 3.7 cm and spanned the entire observed range. This was an order of magnitude higher, and also more variable, than the average rainfall of 0.29 cm (range = 0 to 1.1 cm) in the dry season (April to September). Analysis of transformed rainfall data indicated that this difference was significant (Welch’s *t* test, *P* = 0.0143; Fig. S1 in the supplemental material). Average flow from the Mānoa-Pālolo Stream varied from 0.06 to 17 m^3^ · s^−1^ on sampling days and was strongly correlated with the prior 24-h rainfall in Mānoa Valley (*r* = 0.87, *n* = 13, *P* = <0.0001; Table S2 in the supplemental material). The canal-wide average salinity and temperature for the monthly samplings (*n* = 13) both had significant negative correlations with the prior 24-h rainfall (*r* = −0.84, *P* = 0.0003 and *r* = −0.86, *P* = 0.0002, respectively).

Surface water salinities in the Ala Wai Canal varied from 1 to 36 ppt (mean of 24 ppt) and temperatures from 19.2 to 31.8°C (mean of 27°C; Table 1). Salinity was highly variable throughout the study area, reaching maxima of ≥29 ppt at every site and minima of ≤5 ppt at least once at each site except Site 15, which was the most seaward site in the harbor (minimum salinity of 11 ppt). Consequently, there was no significant difference in average salinity among sites [analysis of variance (ANOVA), *P* > 0.07]. When samples were clustered by general location, average salinities in the upper and lower canal were not significantly different (*P* = 0.7818), but the combined stream mouth sites had a significantly lower salinity on average than either the upper (*P* = 0.0083) or lower (*P* = 0.0016) canal sites.

**TABLE 1:** Variables measured for individual samples (reported to two significant digits)^*a*^

Variable	*N*	Geomean	Mean	Median	SD	Min	Max
*T* (°C)	242	27	27	27	2.6	19	32
Salinity (ppt)	243	20	24	28	9.7	1	36
Nitrate (μM)	209	18	37	17	48	0.02	260
Nitrite (μM)	211	0.47	0.59	0.47	0.47	0.04	3.3
Ammonia (μM)	207	5.5	6.7	5.7	4.3	0.94	22
Phosphate (μM)	211	1	1.3	0.95	1.1	0.2	8.7
Silica (μM)	211	110	140	120	92	11	490
Particulate carbon (μM)	199	130	250	120	650	20	7,500
Particulate nitrogen (μM)	199	16	26	14	51	2.6	560
Chlorophyll^*a*^ (μg · L^−1^)	194	7.6	19	7.3	43	0.4	500
Total bacteria (10^9^ cells · L^−1^)	219	4.2	4.8	4.7	2.3	0.47	11
CaV blue^*b*^ (CFU · mL^−1^)	59	130	400	100	770	12	3,904
*vvhA* gene^*c*^ (copies · mL^−1^)	239	69	330	60	1200	3.4	14,000

a *N*, number of samples measured; Geomean, geometric mean; SD, standard deviation; Min, minimum value; Max, maximum value.

b Culture-based blue CFU when plated on CHROMagar Vibrio medium (CaV).

c qPCR-based estimates.

**FIG 2:**
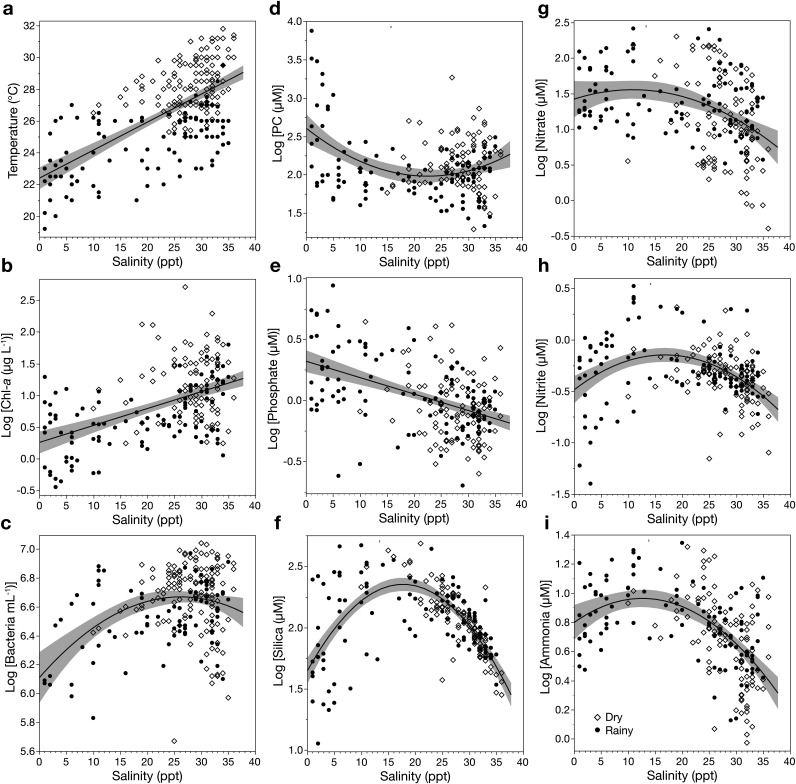
Variability in measured biological and chemical properties of samples as a function of salinity in samples from rainy (solid circles) and dry (open diamonds) seasons. Regressions against salinity are shown for (a) temperature (*r*^2^ = 0.42), (b) log(Chl *a*) (r^2^ = 0.24), (c) log(bacteria) (*r*^2^ = 0.14), (d) log(PC) (*r*^2^ = 0.13), (e) log(phosphate) (*r*^2^ = 0.22), (f) log(silica) (*r*^2^ = 0.463), (g) log(nitrate) (*r*^2^ = 0.13), (h) log(nitrite) (*r*^2^ = 0.152), and (i) log(ammonia) (*r*^2^ = 0.29). Regression lines and 95% confidence limits were fit using only first-order terms unless addition of a quadratic term substantially improved *r*^2^ or reduced root-mean-square error (RMSE). All fits were significant (*P* < 0.0001).

All the measured variables (Table 1) except silica and nitrite displayed overall significant positive or negative significant correlations with salinity (see Table S3 in the supplemental material), but the correlation coefficients were low in many cases because of nonlinearity in the relationships, as illustrated by regression analyses (Fig. 2). Temperature displayed a significant positive linear correlation with salinity (*r* = 0.65, *n* = 242, *P* < 0.0001; Fig. 2a). Correlation and regression analyses for all other variables versus salinity are reported for log-transformed data. Concentrations of Chl *a* (range = 0.4 to 512 μg · L^−1^) showed a significant positive linear (Fig. 2b) correlation with salinity (*r* = 0.49, *n* = 194, *P* < 0.0001). Concentrations of total bacteria (range = 0.47 × 10^6^ to 11 × 10^6^ mL^−1^) also showed significant positive correlations with salinity (*r* = 0.29, *n* = 219; *P* < 0.0001), but the relationship was nonlinear (Fig. 2c). Particulate carbon (PC) (range = 15 to 5,600 μM) had a nonlinear relationship with salinity (Fig. 2d) that resulted in an overall weak but significant negative correlation (*r* = −0.25, *n* = 199, *P* = 0.0003).

Of the dissolved inorganic nutrients, only phosphate (range = 0.2 to 8.7 μM) had a linear relationship with salinity (Fig. 2e) and displayed a significant negative correlation (*r* = −0.46, *n* = 211, *P* < 0.0001). Concentrations of silica (11 to 490 μM), nitrate (0.02 to 260 μM), nitrite (0.04 to 3.3 μM), and ammonia (0.94 to 22 μM) all displayed significant, nonlinear relationships with salinity (Fig. 2f to i), with the highest values occurring at moderate salinities. Despite the nonlinear relationships, there were significant negative correlations between salinity and either nitrate (*r* = −0.32, *P* < 0.0001) or ammonia (*r* = −0.44, *P* < 0.0001). Silica and nitrite, on the other hand, showed highly significant, nonlinear relationships with salinity (Fig. 2f and h) that resulted in low and insignificant correlation coefficients (see Table S3 in supplemental material for the correlation and partial correlation matrix for all variables).

When sites were clustered by location, most nutrients (nitrate, ammonia, phosphate, and silica, but not nitrite), particulate carbon, Chl *a*, and total bacteria were all significantly higher (*P* < 0.01) in the upper canal sites than in the lower canal sites.

### Temporal and spatial variability of *V*. *vulnificus*.

**FIG 3:**
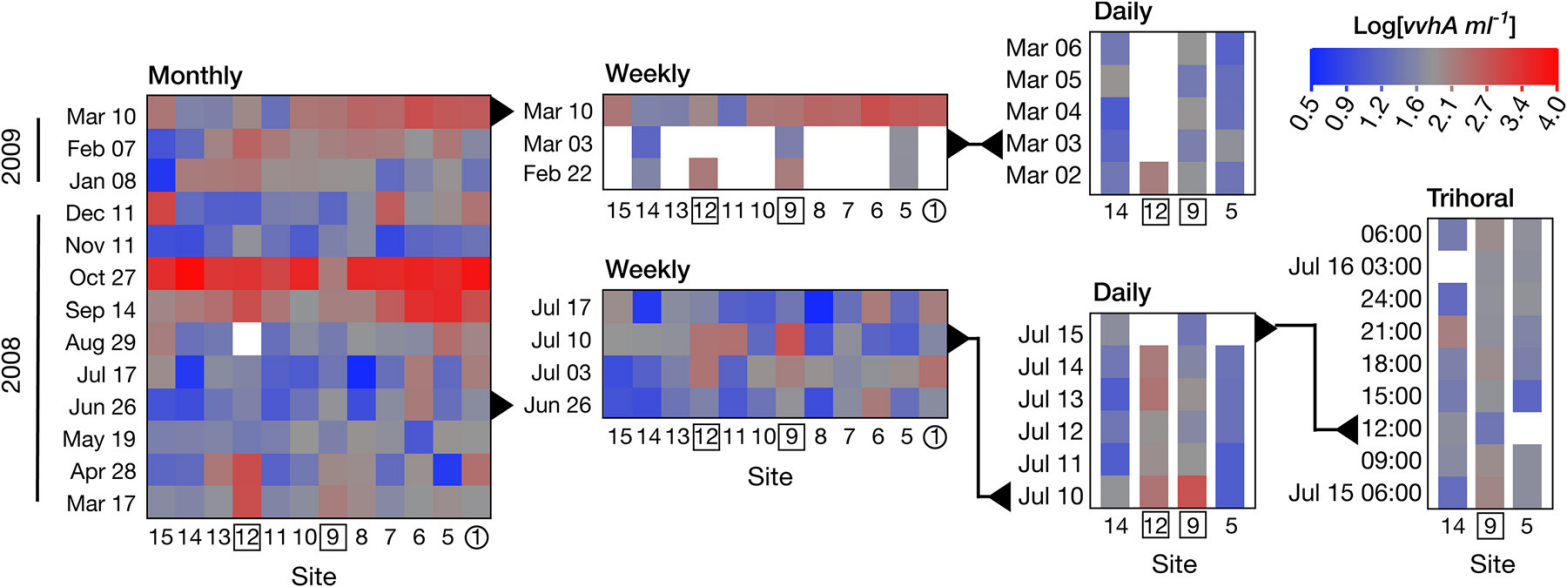
Heat maps illustrating spatial and temporal variability in V. vulnificus. Log(*vvhA*) concentrations are color-coded at each station over time for monthly, weekly, daily, and trihoral sampling events. Overall average log(*vvha*) from all samplings of 1.8 is shown in gray. Concentrations above average are in red and those below average are in blue. Samplings on different time scales are nested and events that overlap in the different graphs are indicated with black triangles and lines. Numbers for sites next to point sources of freshwater are indicated by numbers in squares (Mānoa-Pālolo and Makiki streams) or in a circle (storm drain).

Concentrations of the *vvhA* gene (a proxy for V. vulnificus abundance) varied over several orders of magnitude in space and over time (Fig. 3), from 3 to 13,700 mL^−1^, with an overall geometric mean concentration for all samplings of 68 mL^−1^ (*n* = 239; Table 1). Concentrations of *vvhA* at any given site were highly variable over time, with values above or below average occurring at some point at every location. Although spatial and temporal variability were low during the trihoral sampling over the course of 1 day in July, larger variations were seen over daily or longer time scales. The most dramatic variation was the change from above- to below-average concentrations at every site over the span of 15 days (27 October to 11 November 2008).

Despite the high variability, average log-transformed *vvhA* concentrations for monthly samplings during the rainy season (2.06 ± 0.77, *n* = 84) were significantly higher (Welch′s *t* test, *P* = 0.0065) than those during the dry season (1.75 ± 0.60, *n* = 71; Fig. S2 in the supplemental material). None of the individual sites had an annual average *vvhA* concentration that was significantly different from those of any others (ANOVA, *post hoc* Tukey, *P* ≥ 0.63). However, excluding the stream mouth sites, the annual average concentration of log-transformed *vvhA* for the five sites in the upper canal (2.05 ± 0.74) was significantly higher (*n* = 65 at each site; unpaired *t* test, *P* = 0.0110) than the annual average for five sites in the lower canal (1.75 ± 0.70).

### Relationship of *V. vulnificus* to temperature and salinity.

**FIG 4:**
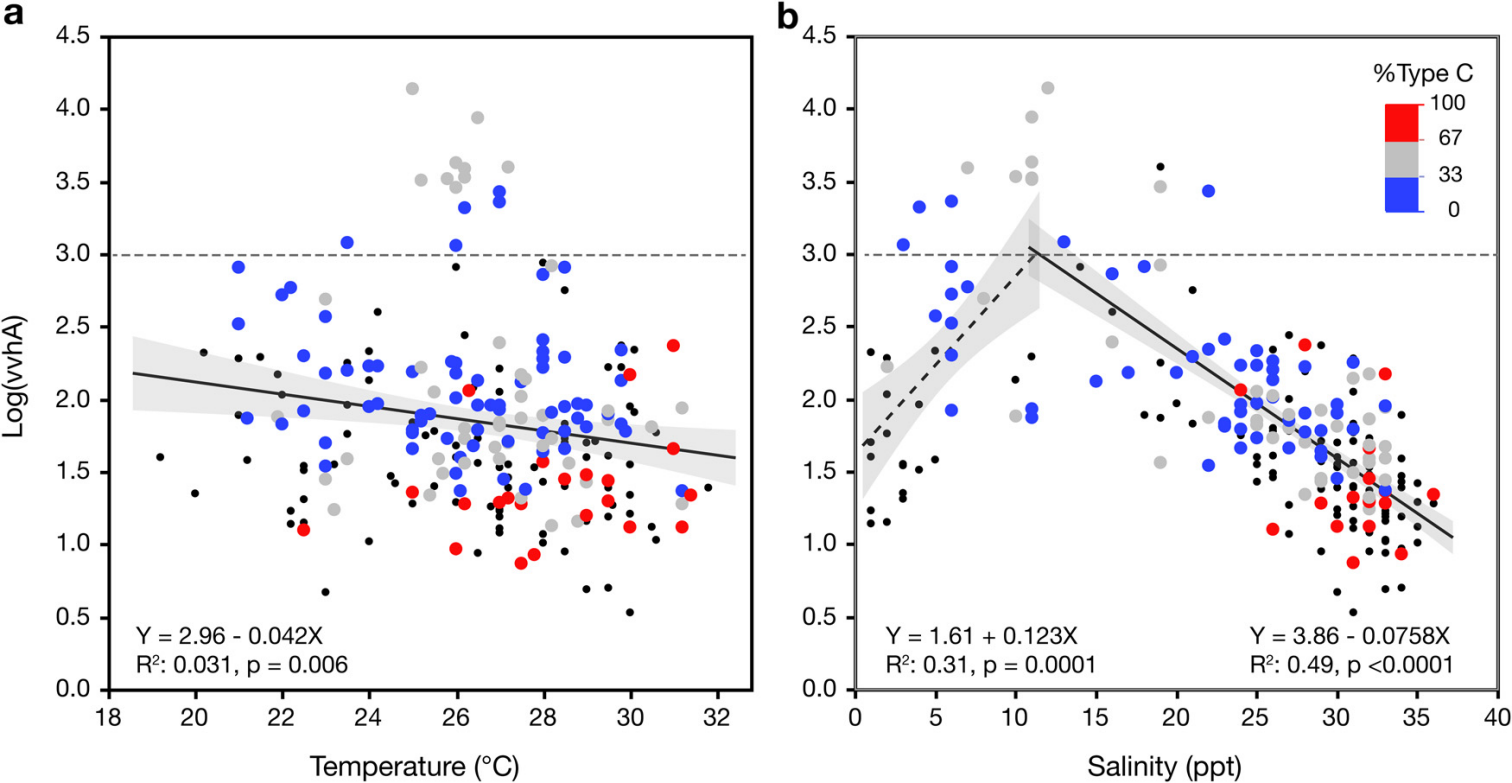
Concentration of *vvhA* as a function of (a) temperature or (b) salinity. Percentage of total *vvhA* that was derived from “C-type” V. vulnificus was determined as the ratio of *vcgC* (C-type) and *vvhA* (total V. vulnificus) gene concentrations and is indicated by the color scale. Blue dots are samples dominated by E-type; red dots are those dominated by C-type. Smaller black dots are samples for which the %C-type could not be determined because of missing data.

Log-transformed concentrations of *vvhA* displayed a weak but significant negative correlation (*r* = −0.17, *P* = 0.0071) with temperature (Table S3 in the supplemental material; Fig. 4a). However, partial correlation analysis indicates that the relationship between log(*vvhA*) and temperature is weakly positive, and significant (*r* = 0.25, *P* < 0.0001) when accounting for the effect of salinity and other variables. There was an overall significant negative correlation between log(*vvhA*) and salinity (*r* = −0.51, *P* < 0.0001; Table S3 in the supplemental material), but linear correlation obscures the relationship between these variables. The maximum *vvhA* concentration was observed at a salinity of 12 ppt, and separate linear regression analyses for samples with salinities of either <12 or ≥12 ppt showed that *vvhA* increased significantly (*r*^2^ = 0.315; F test, *P* = 0.0001) as a function of salinity over the lower range and decreased significantly (*r*^2^ = 0.492; F test, *P* < 0.0001) over the higher range (Fig. 4b).

Concentrations of the *vcgC* gene, indicative of clinical, or C-type, V. vulnificus, were <50% of the *vvhA* gene concentration (total V. vulnificus) in the majority of samples (*n* = 97 of 142) for which data for both genes were available. This suggests that communities were most often dominated by E-type. Both the total and C-type V. vulnificus were most abundant at moderate salinities and declined as a function of salinity, but mean C-type declined at a lower rate. As a result, the contribution of C-type tended to increase as a function of salinity. Samples in which C-type accounted for more than two-thirds of the total (*n* = 23) were only observed in higher-salinity waters (Fig. 4b). Analysis of transformed data suggest that the percentage of C-type was significantly higher (Welch’s *t* test; *P* < 0.0001) in higher-salinity samples (≥25 ppt) than in samples with lower salinity (<25 ppt; Fig. S3 in the supplemental material).

### Relationship between *V. vulnificus* and additional variables.

**FIG 5:**
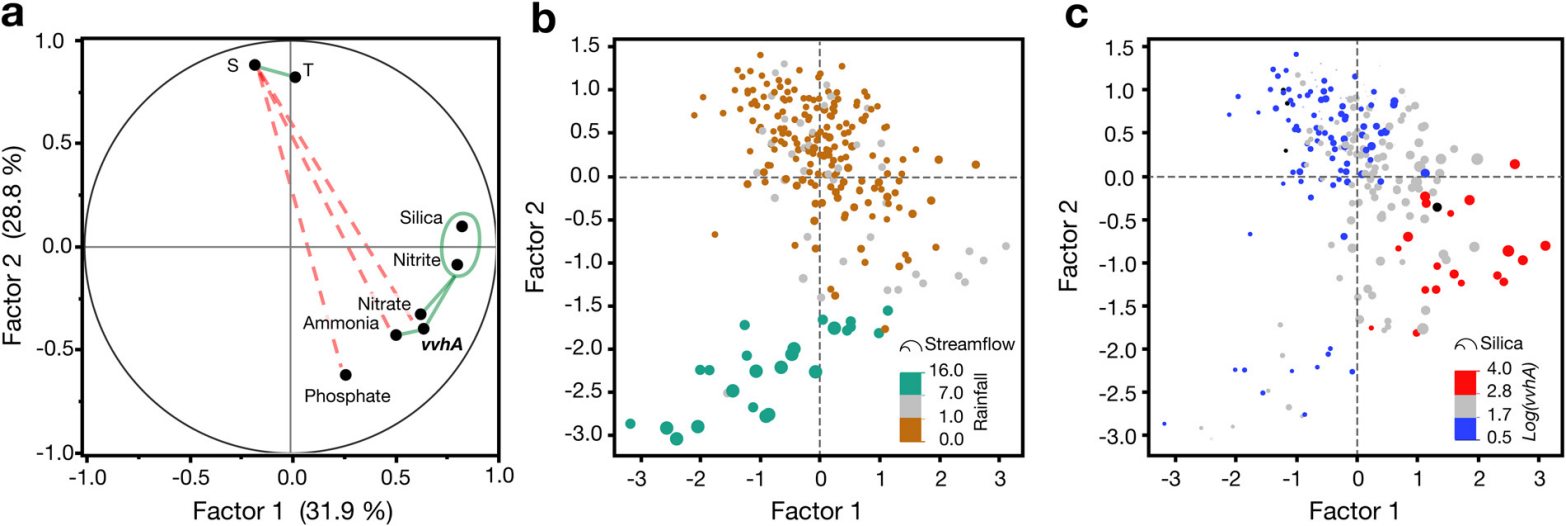
Factor analysis for *vvhA*, temperature, salinity, and nutrients. (a) The factor loading plot for factors 1 and 2 (eigenvalues of >1). Variables with a strong positive correlation (*r* ≥ 0.4) are connected by green solid lines and those with a strong negative correlation (*r* ≤ 0.4) are connected by dashed red lines (b) Plot of factor scores with data points colored by 24-h antecedent rainfall in Mānoa Valley (cm) and scaled in size so that area is proportional to streamflow in the Mānoa-Pālolo Stream. (c) Plot of factor scores with data points colored according to log(*vvhA*) concentration and scaled in size so that area is proportional to silica concentration.

To understand additional factors that may be important in controlling V. vulnificus in this habitat, factor analysis was conducted with *vvhA*, temperature, salinity, and nutrient data (Fig. 5a). Two factors had eigenvalues of >1. The strongest positive correlations (*r* ≥ 0.4) were between *vvhA* and silica or reduced nitrogen species, which were associated with Factor 1, and strong negative correlations (*r* ≤ −0.4) were found between salinity and *vvhA*, ammonia, and phosphate along Factor 2. Plots of the factor loading values with points coded by rainfall and streamflow (Fig. 5b) illustrate the relationship between indicators of freshwater input and salinity along the Factor 2 axis. Coding the points by log *vvhA* concentration and silica concentration illustrates the association of these variables (along with reduced nitrogen species) with Factor 1. Overall, the highest concentrations of *vvhA* occurred when rainfall in the valley was moderate, but streamflow was relatively low, and concentrations of silica were elevated.

**FIG 6:**
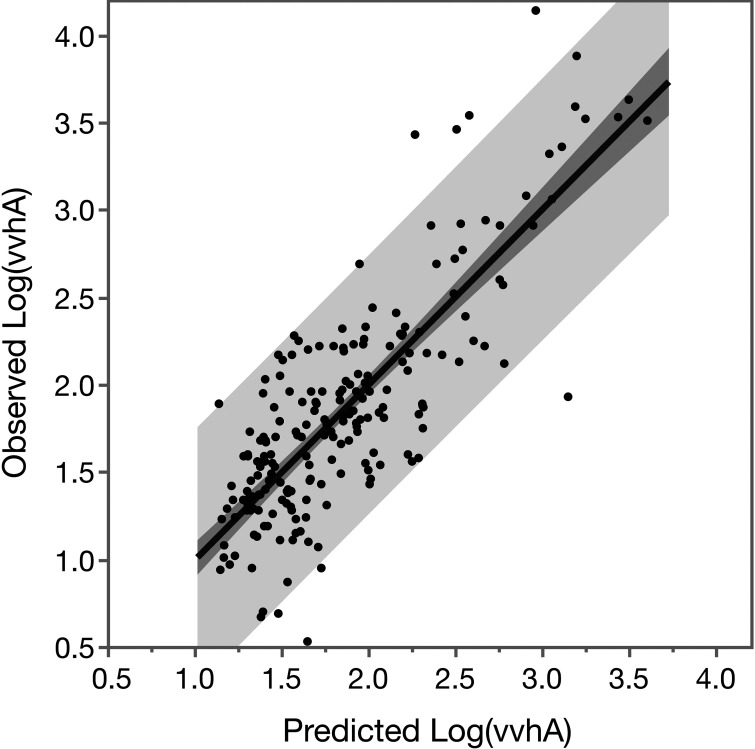
Observed versus predicted values of log-transformed *vvhA* gene copies per mL. Predicted values are combined from two separate models (one for samples with <12 ppt, one for those with ≥12 ppt). Predicted values are restricted to individual samples for which all predictor variables were measured within a given salinity range (*n* = 204 out of 239 in total). Darker and lighter shading illustrates 95% confidence limits of the fit and prediction, respectively. Combined, the models explain a significant amount of the variation in the observations: *r*^2^ = 0.661, RMSE = 0.37, F test (1, 204) = 396.90, *P* < 0.0001.

Generalized regression models for predicting *vvhA* concentrations over the two different salinity ranges were generated using either the overall best subset (<12-ppt model) or the best subset with the minimum number of variables (≥12-ppt model). Only properties intrinsic to individual samples were included in this analysis (i.e., tides, rainfall, and streamflow were not considered). For samples with salinities of <12 ppt (*n* = 39 out of 41 samples because of missing nutrient data), a subset of 4 (temperature, nitrite, silica, and PC) out of 8 variables explained 75% of the observed variation with the following equation:

log(vvhA) = 0.154•T+1.015•log(nitrite)−0.600•log(silica)−0.850•log(PC)+2.170

where *T* is temperature in °C, and nitrite, silica, and particulate carbon (PC) are in units of μM (model fit illustrated in Fig. S4a in the supplemental material). For samples with salinities of ≥12 (*n* = 163 out of 198 possible samples because of missing nutrient data) a subset of just 3 (temperature, salinity, and phosphate) out of 7 variables explained 55% of the variability:

log(vvhA) = 0.0360•T−0.0727•S+0.515•log(phosphate)+2.835

where *T* is temperature in °C, *S* is salinity in units of ppt, and phosphate is in units of μM (model fit illustrated in Fig. S4b in the supplemental material). PC was removed prior to variable selection in the latter model because initial analysis showed that it offered no significant explanatory power at salinities of >12 ppt, and missing data would have further restricted the samples included in the analysis. When predictions from the two models were combined, 66% of the variability in log(*vvhA*) over the entire salinity range was explained overall (Fig. 6).

Models in which either a quadratic term for salinity or the derived variable ΔSal_opt_ were included explained similar amounts of variability (*r*^2^ = 0.61 and 0.63, respectively; *P ≤ *0.0001) using different sets of five variables (Fig. S5 in the supplemental material), but were slightly outperformed by the combined models above.

### System-wide controls on *V*. *vulnificus*.

**FIG 7:**
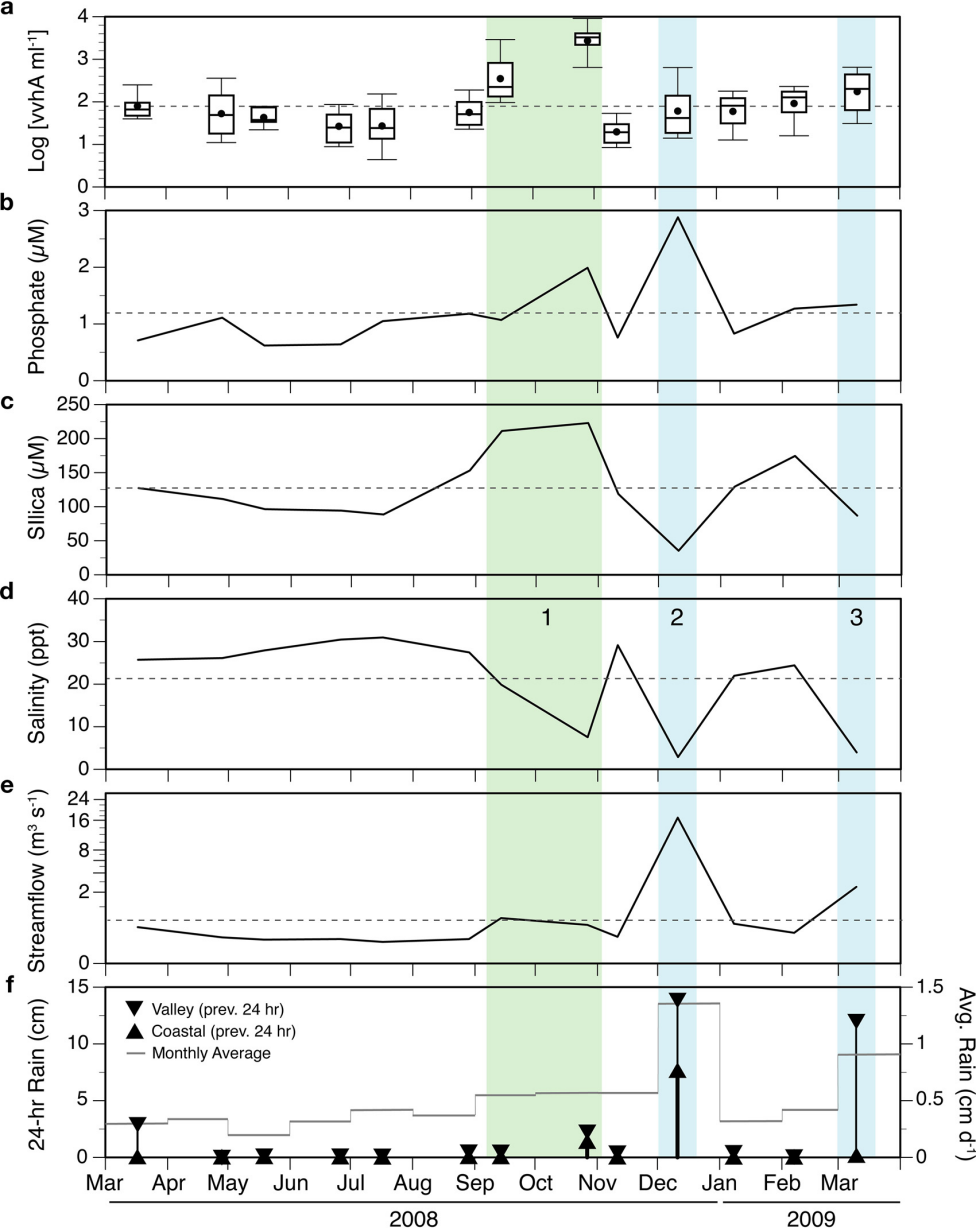
Time series of variables in or influencing the Ala Wai Canal system. Shown are (a) variations of *vvhA* concentrations as box plots of all log-transformed values measured at every site at each monthly sampling and canal-wide geometric means of (b) phosphate, (c) salinity, (d) silica, (e) average streamflow in the Mānoa-Pālolo stream on the day of sampling, and (f) rainfall in the 24-h period preceding sampling as measured at the Honolulu coastal (upward triangles) and Mānoa Valley rain gauges (downward triangles). The axis for streamflow in panel e is on a cube-root scale to show more detail at low flow rates. Daily rainfall average for the month is shown as the mean for both sites (gray line). Dashed lines in panels a to e show mean values over the time period. Three periods of freshening (shaded areas, numbered 1 to 3) were observed either without (green shading) or with (blue shading) accompanying high rainfall and above average streamflow.

To smooth out inter-station variability and focus on temporal variations in *vvhA*, canal-wide averages for the variables for each monthly sampling were also analyzed in relation to system-wide drivers of rainfall and streamflow (Fig. 7). In general, average rainfall, streamflow, phosphate, silica, and *vvhA* were all below average, and salinity above average, during most of the dry season, with minimal variability. During the rainy season, periodic heavy rainfall resulted in high variability, with excursions in all variables well above and below their overall averages.

Three freshening events are evident from dips in the average salinity in the canal during the rainy season (Fig. 7). The first begins in September and peaks in October 2008 following increases in rainfall and streamflow. The average monthly rainfall increased from ≤0.75 cm · d^−1^ in the preceding months to 1.0 cm · d^−1^ from September to October, and the 24-h antecedent rainfall for the October sampling was 2 cm (up from ≤0.5 cm in other samplings). Streamflow increased from an average of 0.07 m^3^ · s^−1^ for the July and August samplings to an average of 0.4 m^3^ · s^−1^ for September and October. In response, canal-wide average salinity dropped to 8 ppt and average silica concentrations in September and October reached their highest concentrations (223 to 244 μM). Phosphate displayed only a small local peak in average concentration (2 μM). Canal-wide average concentrations of *vvhA* reached a maximum during this event, from 350 (range = 67 to 3,500) gene copies · mL^−1^ in September to an average of 2,700 (range = 170 to 13,700) gene copies · mL^−1^ in October. The average concentration in October was significantly higher than that at any other monthly sampling (ANOVA, *post hoc* Tukey, *P ≤ *0.0005). At the subsequent sampling 15 days later (November), rainfall had stopped, streamflow, phosphate and silica had declined, average salinity had increased to 29 ppt, and *vvhA* was at the lowest average concentration of the study, with an average of 20 (range 7 to 63) gene copies · mL^−1^ across all sites.

A second, more pronounced drop in salinity occurred in December 2008 in response to heavy rainfall recorded at both the coastal and Mānoa Valley rain gauges, resulting in the highest recorded streamflow (17 m^3^ · s^−1^), minima in salinity (3 ppt) and silica (34 μM), and the highest average phosphate concentration (2.9 μM). In contrast to the previous freshening event, *vvhA* was not significantly elevated (61 gene copies · mL^−1^) and was near the overall study average.

A third freshening event occurred at the time of the last sampling in March 2009, because of heavy rainfall in Mānoa Valley, but not at the coast. Streamflow (2.5 · m^3^ s^−1^) was above average and intermediate between the first and second events, and salinity was again significantly reduced (4 ppt). The effects on phosphate (1.3 μM) and silica (87 μM) were modest, with phosphate being just above the long-term average and silica just below. The mean concentration of *vvhA* reached its third highest level at this time, reaching 175 (range = 22 to 811) gene copies · mL^−1^ after steadily increasing each month from the lowest value in November.

**FIG 8:**
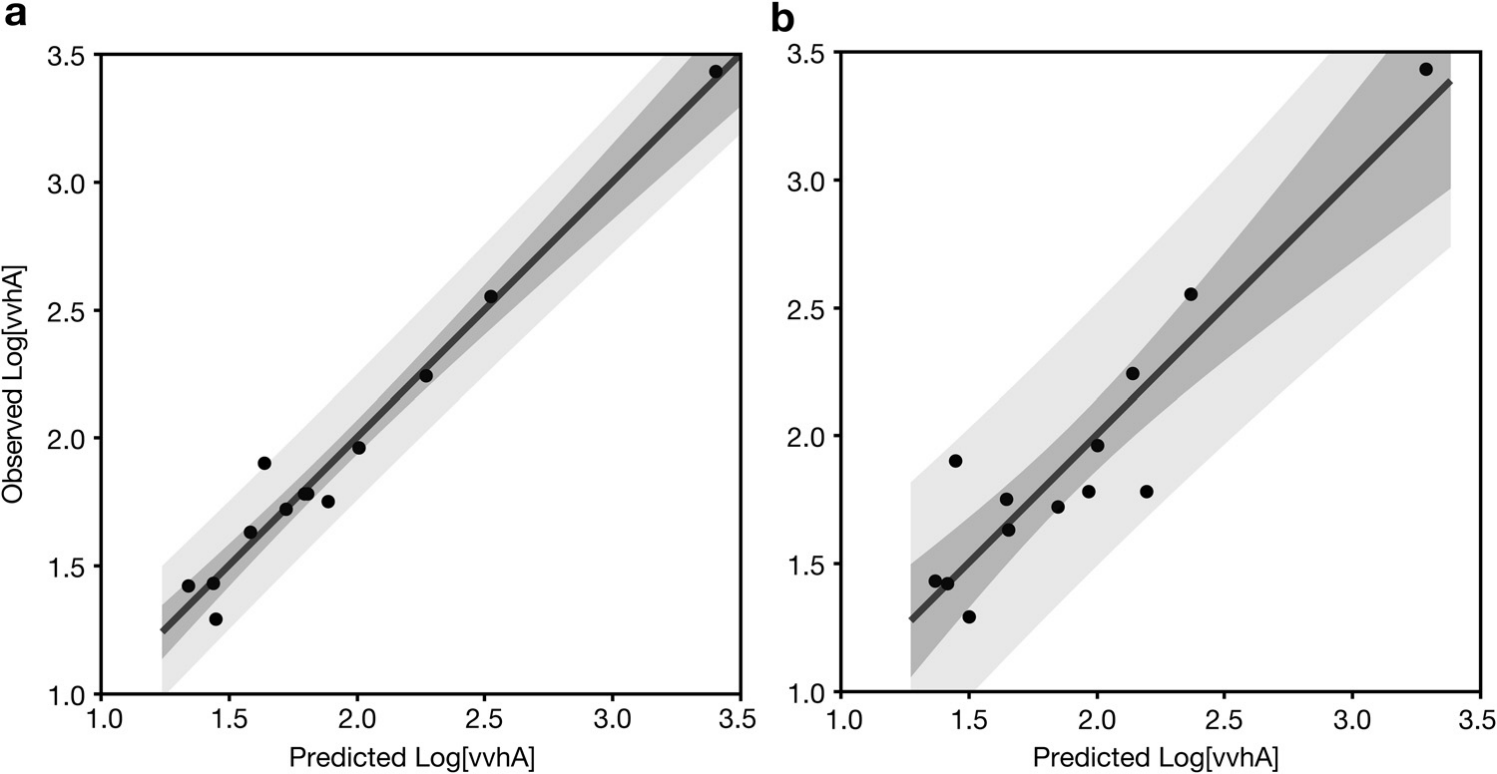
Observed versus predicted canal-wide averages of log-transformed *vvhA* concentrations. Predictions are derived from (a) the best subset of variables (salinity, silica, streamflow, particulate carbon) from generalized regression model (*r*^2^ = 0.97; RMSE = 0.11; F test *P* <  0.0001) or (b) a restricted subset of two variables (rainfall and salinity) that are easily measured autonomously (*r*^2^ = 0.86; RMSE = 0.22; F test *P* < .0001). Darker and lighter shading illustrates the 95% confidence limits of the fit and prediction, respectively.

Multiple linear regression was used to determine which subset of variables best predicted canal-wide average log(*vvhA*) concentrations over the year. The model resulting from the best subset out of all combinations of 12 possible variables was:

$$\text{log}{\left(\mathrm{vvhA}\right)}_{\mathrm{avg}}=-1.125•{\text{streamflow}}^{1/3}-0.07633•\text{salinity}+0.00502•\text{silica}+0.00151•\text{PC}+3.522$$

where streamflow is in units of m^3^ · s^−1^ and salinity, silica, and particulate carbon (PC) are in units of μM. All variables are the geometric means for all sites in the canal for each monthly sampling (*n* = 13). Linear regression of observed versus predicted *vvhA* suggests that 97% of the canal-wide average variation in *vvhA* could be explained with the selected variables (Fig. 8a).

A second, simpler model using a minimum of readily measurable variables (salinity and rainfall) was also constructed:

$$\text{log}{\left(\mathrm{vvhA}\right)}_{\mathrm{avg}}=\;-0.162•\text{rainfall }-0.0956•\text{salinity}+4.348$$

where rainfall is average rainfall in cm for the prior 24 h at the Mānoa Valley gauge, and salinity is canal-wide average salinity in ppt. This simpler model explained 83% of the variability in average log-transformed concentrations of *vvhA* (Fig. 8b).

## DISCUSSION

### Temporal and spatial variability of *V*. *vulnificus*.

V. vulnificus, as inferred from *vvhA* gene, was consistently detected throughout the year in the Ala Wai Canal and Harbor system but varied dramatically over space and time. Sampling on different temporal scales showed minimal variation in V. vulnificus within a day, but dramatic and stochastic variations on longer time scales and among sites. This suggests that factors with regular intra-day variations (e.g., tides, or daily changes in temperature and primary productivity driven by insolation) had relatively little influence on V. vulnificus concentrations. The largest absolute change in canal-wide average *vvhA* concentrations seen over the entire study occurred in a span of 2 weeks. The observation that V. vulnificus concentrations were higher on average in the rainy versus the dry season, yet the lowest average concentration recorded in the study also occurred in the rainy season within weeks of the highest abundances, suggests that freshwater input, which occurs stochastically but with an underlying strong seasonal component, is the most significant contributor to variability in V. vulnificus abundance in this environment.

These results support an earlier hypothesis (22) that in tropical and some subtropical climates, where the temperature range is narrow and persistently warm, salinity is a stronger determinant than temperature of V. vulnificus abundance. This is consistent with the seasonal variation in V. vulnificus in oysters in India, which is not related to temperature but to summer monsoonal rains lowering salinity (36). In Hawaiʻi, with its rainy season in winter months, there is thus a tendency toward a seasonal cycle in V. vulnificus abundance that is inverted from the pronounced temperature-driven cycle found at higher latitudes and the monsoon-driven cycle in India.

### Variable sources and influence of freshwater inputs.

The two major sources of freshwater to the Ala Wai Canal are surface runoff (primarily point source from streams and storm drains) and groundwater seeps. Compared to surface runoff, groundwater in Hawaiʻi tends to be enriched in silica due to prolonged water-rock interactions (37) and depleted in phosphate due to interactions with lateritic soils containing high concentrations of iron and aluminum oxyhydroxides (37, 38). These differences, along with information on rainfall and streamflow, are helpful in identifying the primary source of the freshwater entering the canal. In the factor analysis, Factor 1 may be interpreted as a latent variable for groundwater (high loading for silica, but low for phosphate), and Factor 2 as a latent variable for (negative) surface runoff (high, but opposing, loading of salinity and phosphate). Plots of the loading scores reinforce the observation that V. vulnificus tended to be highest at moderate salinities, and suggest that groundwater was a relatively more important source of freshwater input under those conditions (low streamflow, but elevated silica). When rainfall was highest, surface runoff contributed more to freshwater input (highest streamflow with high phosphate, low silica) and was associated with lower concentrations of *vvhA*.

This variable relationship between *vvhA* and freshwater source was also discernible in the temporal changes in variables when averaged across all canal sites. Of the three major freshening events, the first, with relatively high silica and low phosphate, suggests a significant contribution from groundwater. This is consistent with the observation of significant drops in salinity despite only modest increases in streamflow compared to the summer months. This presumed increase in groundwater input appears to have been driven by a moderate increase in monthly average rainfall in both September and October, coupled with a modest increase in average rainfall during the 24 h preceding the sampling which was greater at higher elevations in the watershed than locally.

The second freshening event, with a high concentration of phosphate but low silica, appears to be dominated by surface runoff, resulted from a Kona storm on the south shore of O'ahu (39). A Kona storm is a rain event that deviates from the normal northeasterly trade-wind driven patterns that govern Hawaii’s weather, and occurs when southwestern Kona winds bring heavy rains to the southern shore of O'ahu. This storm resulted in unusually high rainfall, higher both in the watershed and locally, during the 24 h prior to sampling.

The third freshening event on 10 March 2009 appears to have a source signature which is intermediate to the two prior events in terms of stream flow and silica. This is consistent with an average rainfall in the preceding 24 h in the watershed that was high enough to increase downstream runoff and groundwater discharge into the canal (as in the previous event), but with limited local precipitation that, unlike the previous event, did not contribute appreciably to surface runoff.

The concentrations of *vvhA* during these three events suggest that at least the magnitude, if not the sources, of the freshwater input to the canal has a large influence on V. vulnificus abundance. The mixing of freshwater with seawater in the canal is expected to have competing influences on V. vulnificus, because it simultaneously alters temperature, salinity, and residence time. At sustained, moderate levels of freshwater input (such as that from ground water intrusion driven by moderate rainfall higher in the watershed), both the temperature drop and the decrease in residence time are relatively small, but the freshening is sufficient to result in salinities that are optimal for V. vulnificus, thus explaining the unusually high abundance of V. vulnificus in September and October 2008. During unusually intense storms, especially with high rainfall lower in the watershed (December 2008), the very high levels of surface runoff appear to suppress V. vulnificus abundances in the canal. This is likely a result of a simultaneous reduction in growth rate (caused by decreases in both temperature and salinity to below optimum) and a reduction in the residence time of water in the canal that accompanies increases in streamflow (40). It is possible that the properties of groundwater (e.g., micronutrient concentrations) also specifically promote growth of V. vulnificus, but we have no evidence for this at present.

Although intense storms can temporarily suppress canal-wide average concentrations of V. vulnificus in the canal/harbor system, the actual changes are site-specific. For example, we observed that during the December 2008 storm, V. vulnificus abundance, despite a lower canal-wide average, was higher than average at Site 15, the most seaward site in the Ala Wai Boat Harbor. In this location, salinity was temporarily reduced to 13 ppt (in the optimal range for V. vulnificus) compared to the typical average for this site of ≥30 ppt (39). Salinity remained below the average in the harbor for 16 h following the cessation of rainfall. This suggests that the sites posing the highest risk of infection by V. vulnificus will vary depending on rainfall patterns, and can even include the harbor, which usually had some of the lowest concentrations. This condition-dependent elevated risk in the harbor is consistent with an unfortunate incident of the infection and death of an individual whose open wounds were exposed to harbor water following a long period of intense rainfall (41).

### Patterns of *V*. *vulnificus* strain abundance.

C-type V. vulnificus strains are the strains most frequently associated with infections in humans (10), but they are often less abundant than E-type strains in environmental samples (10, 42). This appeared to be the case at our study site as well, but determining an accurate overall mean was complicated by differences in assay sensitivities. The standard curves for both the *vvhA* and *vcgC* assays had high reproducibility (see Fig. S6 in the supplemental material), but the latter assay was less sensitive (a two-cycle difference for detecting equivalent copy numbers). The lower sensitivity for *vcgC*, coupled with lower concentrations of C-type on average, resulted in *vcgC* being below the detection limit in a greater proportion of samples (28%) compared to *vvhA* (2.5%) making the overall mean %C-type uncertain.

Because %C-type is calculated as a ratio, the variance in this derived number also increased as total V. vulnificus concentrations declined, which could inflate the mean percentage. However, by restricting the analysis to data in which both genes were detected with confidence and accounting for unequal variance, we still found a significantly higher %C-type in more saline waters, which is consistent with some previous observations. Williams et al. (42), for example, observed a negative influence of salinity on the abundance of E-type and C-type strains, but the effect was greater for E-type. Lin and Schwarz (43) observed that when temperature decreased and salinity increased, *in situ* abundance of 16S rRNA A-type strains (analogous to E-type) decreased while B-type (analogous to C-type) increased and temporarily became the dominant genotype. C-type V. vulnificus also dominated in oysters in this area when salinities and temperatures were higher (44). Other studies in high-salinity (>32 ppt) coastal waters have found that either a majority (8) or all (45) of the isolates obtained were of B-type (C-Type). These observations support the contention that these different genotypes reflect distinct ecotypes, with the C-type having greater stress tolerance (11).

The importance of distinguishing the C-type when assessing exposure risks in recreational waters is not clear. Both genotypes cause infections in humans and either type can be lethal in mouse models (46). Comparison of the 16 lethal strains in that study indicates, however, that the average percent mortality was significantly lower for E-type than for C-type (*n* = 8 for each type; *t* test, *P* = 0.0028). On the other hand, E-type has been reported to be much more strongly associated with wound infections than C-type, and the latter is more associated with infections caused by ingestion (7). Consequently, E-type may be of particular concern in recreational waters that are not shellfish harvesting areas. Considering that either V. vulnificus genotype can cause serious infection, and that our data for *vvhA* are more complete and robust than those for *vcgC*, we focused our statistical modeling efforts on understanding the evironmental influences on total V. vulnificus.

Multiple linear regression analysis was used to model V. vulnificus abundance using a reduced number of variables. Although these variables explained a significant percentage of the variability in V. vulnificus abundance, a great deal of sample-to-sample variability remains unexplained, which is not uncommon (22, 28). All of models using individual samples also tended to underpredict the highest concentrations of *vvhA*. Predicting system-wide average concentrations of V. vulnificus, on the other hand, was much more successful. A model with the best subset of four variables explained 97% of the variability, and a much simpler model relying on only two readily obtainable measurements (rainfall and salinity) still accounted for much of the variability and might prove more useful in practice for predicting relative risk of V. vulnificus exposure from the canal and harbor waters.

The high level of predictability for system-wide average V. vulnificus is similar to that achieved using logistic regression to predict *vvhA* as a binary response variable, either as presence or absence (47) or as low versus high abundance (48). Improvements in the prediction of V. vulnificus at higher resolution may be realized by combining biological population models for V. vulnificus with physical models of coastal circulation (48). In the meantime, the results from this study provide a detailed description of the ecology of V. vulnificus in tropical estuarine waters of Hawaiʻi. The results are a useful first step toward predicting and, ultimately taking steps to mitigate, the incidence of V. vulnificus infections.

## MATERIALS AND METHODS

### Study site.

Sampling took place in the Ala Wai Canal (Fig. 1), a 3.1-km long engineered waterway located on the southern coast of Oʻahu which separates Waikīkī and urban Honolulu (49). A watershed that covers 42.4 km^2^ drains into the Ala Wai Canal via the Mānoa and Pālolo streams, which merge to form the Mānoa-Pālolo Stream prior to entering the canal, and the Makiki Stream, all of which run through urban areas before reaching the canal. Consequently, the streams are contaminated with a variety of anthropogenic substances, and their convergence in the Ala Wai Canal has contributed to its pollution and eutrophication (49, 50). The influx of fresh water from the streams creates a salinity gradient with a typical salt-wedge structure. Tidal flow causes seawater to flow landward on the flood tide and seaward on the ebb tide and remain at depth. The freshwater streams flow seaward on all tides, creating a freshened water surface layer, estimated to extend to an average depth of 0.5 m, which is highly variable both in salinity and thickness (40). Sediments are continually deposited in the canal at the mouth of the Mānoa-Pālolo Stream, causing the build-up of a sill that restricts flushing of deep water in the uppermost section of the canal.

### Sampling locations, dates, and times.

Sampling of the Ala Wai Canal spanned 13 months beginning on 17 March 2008 and concluding on 10 March 2009, covering the nominal dry summer (April to September) and rainy winter (October to March) months. Samples were collected monthly at 12 sites in the Ala Wai Canal, numbered (1 and 5 to 15) by distance from the shallow upper section of the canal (Site 1) to the Ala Wai Harbor (Site 15). Site 9 was just inside the mouth of the Mānoa-Pālolo Stream and Site 12 was at the mouth of the Makiki Stream (Fig. 1; for site coordinates see, Table S1 in the supplemental material). Missing site numbers 2 to 4 referred to other samplings at Site 1 that were not used in this study. Sampling at a higher temporal resolution was also conducted during the dry and rainy seasons to assess changes on shorter time scales. Samples were collected weekly at all sites for 4 weeks from 26 June to 17 July 2008, and again for 3 weeks from 22 February to 10 March 2009. Samples were also collected at a reduced number of sites (Sites 5, 9, 12, 14) daily for 6 days from July 10 to 15, 2008, daily for 5 days from March 2 to 6, 2009, and once every 3 h (trihoral) for 24 hours at Sites 5, 9, and 14 from July 15 to 16, 2008.

### Rainfall and streamflow.

Rainfall data collected by National Weather Service rain gauges (part of the Hawaiʻi Hydronet System) at 15-minute intervals were retrieved from the NWS website (https://www.weather.gov/hfo/hydronet-data). Data from two gauges were selected for analysis. The first was HI-18 (NOAA no. MNLH1), located near the origin of Mānoa Stream (N21.3161 W157.8142) at an elevation of 150 m in Manoa Valley (“Valley” rainfall). The second is HI-26 (ALOH1), located at Aloha Tower (N21.3060 W157.8662) in downtown Honolulu near sea level (15 m) at the coast (“Coastal” rainfall). From these data, average daily rainfall for all sampling months was determined as well as total rainfall from each 24-h period prior to sampling. Data on tidal flux were obtained from the National Ocean Service (https://tidesandcurrents.noaa.gov/noaatidepredictions.html?id=1612340), using tide gauge no. 1612340. Streamflow data were obtained from the United States Geological survey (https://waterdata.usgs.gov/usa/nwis/uv?16247100) for the Mānoa-Pālolo Stream gauge no. 16247100.

### Water sample collection and processing.

Whole-water samples were collected from the top 10 to 30 cm at all sites in acid-washed bottles with a pole sampler, stored on ice (except samples used for culturing, which were kept at ∼15°C with cold packs), and transported to the laboratory within 3 h of collection. Subsamples (ca. 25 mL) for nutrient analysis (*n* = 207 to 211) were frozen and shipped on dry ice to the Oregon State University nutrient analysis facility for determination of dissolved silica, phosphate, nitrate plus nitrite, nitrite, and ammonium concentrations (51). Nutrient concentrations were measured during every sampling event, excluding two weekly sampling events in July 2008 (July 3 and 7). The values for the mean, number of samples, median, minimum, and maximum of the measured nutrients have been previously reported (39).

For particulate carbon (PC) or nitrogen (PN) and chlorophyll *a* (Chl *a*) measurements, subsamples (25 to 200 mL) were filtered onto precombusted glass fiber filters (GF/F, Whatman) in duplicate and stored frozen until analysis. For PC and PN (*n* = 199), filters were pelletized and combusted in a high-temperature combustion CN analyzer, the CE-440 CHN Elemental Analyzer (Exeter Analytical, Inc.), following HOT program protocols (52). Filters for Chl *a* analysis (*n* = 194) were extracted in 100% acetone at −20°C for 7 days. Fluorescences of extracts and standards were measured using a Turner AU10 Fluorometer before and after acidification (53).

Samples for bacteria counts (*n* = 219) were fixed with filtered (0.2 μm) formaldehyde (10% wt/vol final concentration) in a cryovial (Nalgene) and stored at −80°C. Total bacteria were counted by thawing samples, staining with SYBR Green I, and analyzing on an acoustic focusing flow cytometer (Attune; Thermo Fisher Scientific).

Samples for molecular analysis (*n* = 243) were pressure-filtered via peristaltic pump through a 0.22-μm polyethersulfone filter capsule (Sterivex, Millipore), then stored at −80°C until extracted. Most of the samples (94%) were filtered to the target range of 500 to 550 mL, but the volume was smaller for 15 samples (100 to 400 mL) as a function of increasing particulate carbon concentrations which prematurely fouled the filters and drastically reduced flow rate.

### Cultivation on vibrio-selective medium.

For five of the monthly samplings (March, June, September, and December 2008, and March 2009), water samples from every site were assayed for colony counts on a chromogenic, vibrio-selective medium, CHROMagar Vibrio (DRG International). Samples were diluted 10- to 125-fold in sterile peptone water (3% NaCl, 0.15% peptone) and a fixed total volume of 5 mL was filtered immediately through 0.45-μm pore size, mixed cellulose ester filters (47 mm, GN-6; Pall) to achieve plated volumes corresponding to 0.04 to 0.5 mL of the original sample. Filters were placed face-up on the medium and incubated at 37°C. After overnight incubation (12 to 18 h), blue colonies were enumerated as putative V. vulnificus on plates with most appropriate numbers of colonies (ca. 5 to 200 CFU).

### DNA extraction and purification.

DNA was extracted from the Sterivex filters using a Masterpure Nucleic Acid Extraction Kit (Epicentre). A 600-μL volume of Masterpure Tissue and Cell Lysis Solution containing recommended quantities of proteinase K was added to each Sterivex filter. The ends of the filters were sealed, and the filters were incubated on a rotisserie in a hybridization oven at 65°C for 15 min. Fluid was recovered from filter housing by aspiration with a syringe. The filling with buffer, incubation, and buffer recovery steps was repeated twice more and the combined extract from all three rounds was pooled (total volume ca. 1.8 mL). A 300-μL volume of the pooled extract was processed according to the Masterpure kit guidelines and the remainder was archived. Accounting for all the raw extract volume, total DNA yields ranged from 1 to 540 μg · L^−1^ of canal water (geometric mean of 30 μg · L^−1^). Following initial purification, the resuspended DNA (200 μL TE) was passed through a spin column containing acid-washed polyvinylpolypyrrolidone (PVPP) to remove any residual inhibitors (54). DNA concentration in each sample was quantified fluorometrically (Quant-iT Broad Range DNA kit, Life Technologies) both before and after the PVPP purification step, to account for losses incurred during the purification stage (average recovery, 60%). The geometric mean concentration of DNA in the final purified extracts was 7 ng · μL^−1^ (range = 0.1 to 54 ng · μL^−1^). Extracts were stored at −80°C until assayed.

### Quantitative PCR.

Total V. vulnificus was estimated by TaqMan quantitative PCR (qPCR) targeting the hemolysin gene, *vvhA*, using previously published primer and probe sequences (55). Quantification of C-type V. vulnificus used primers and probes targeting the virulence-correlated gene variant, *vcgC* (56). Both assays were prepared as 25-μL reactions with 12.5 μL of TaqMan Universal PCR Master Mix (Applied Biosystems), 1.5 μg · μL^−1^ final concentration of non-acetylated bovine serum albumin (Applied Biosystems) 0.25 to 0.9 μM (each) of the appropriate primers and probe (sequences presented in Table S4 in the supplemental material), 2 to 5 μL of DNA template (equivalent to 0.01 to 2 mL of original sample, after accounting for DNA loss and dilution), and water as needed. For the *vvhA* assay, primers were added at 0.9 μM each and the probe at 0.25 μM. For the *vcgC* assay, primers and probe were each added at 0.5-μM final concentrations. Cycling conditions consisted of initial denaturation at 95°C for 10 min, then 40 cycles of denaturation at 95°C for 15 s, and annealing/extension at 60°C for either 60 s (*vvhA*) or 90s (*vcgC*). All qPCRs were performed in triplicate with the DNA template in the final replicate diluted 10-fold to check for inhibition (57) and with additional replication and dilution (up to 50-fold) performed on samples showing inhibition. The amplified PCR product was detected by monitoring the increase in fluorescence signal generated from the 6-carboxyfluorescein-labeled probe using a Realplex^2^ Mastercycler (Eppendorf). Data were analyzed using Realplex software (Eppendorf) to determine quantification cycle (*C_q_*) values. Standard curves for both assays consisted of serial 10-fold dilutions (500,000 to 5 genome copies per reaction) of genomic DNA from V. vulnificus strain YJ016 (*vvhA*+ and *vcgC*+) assayed in duplicate in each run. The efficiency of amplification, based on the standard curves across independent qPCR runs, ranged from 97 to 104% for *vvhA* (*n* = 11) and from 100 to 110% for *vcgC* (*n* = 11). Standard curve intercepts varied little (<1%) among curves from independent runs of each assay but differed for *vvhA* (40.1 ± 0.3) versus *vcgC* (42.3 ± 0.3) (Fig. S6, Table S5 in the supplemental material).

Reporting limits were based on a maximum cycle number of 38 (equivalent to 4 to 6 gene copies per reaction) for *vvhA* or 39 (8 to 13 gene copies per reaction) for *vcgC*. This translates into concentration reporting limits for the original sample of 2 to 374 gene copies · mL^−1^ for *vvhA* and 4 to 220 for *vcgC*, depending on initial volume filtered, DNA recovery, and extract dilution factor. At least 2 (up to 4) replicate assays inferred to be free of significant inhibition and above the reporting limit were averaged. Out of 243 total qPCR assays for V. vulnificus abundance (*vvhA*), 17 (ca. 7%) had issues that made them unreliable or unavailable (inhibition, below the reporting limit for the assay, or absence of data). In 13 of these instances, abundances were instead inferred from blue colony counts on CHROMagar Vibrio medium based on the strong correlation (*r* = 0.8) between log-transformed concentrations of blue colony counts and *vvhA* gene copy numbers (Fig. S7 in the supplemental material). No corresponding colony counts were available for the remaining samples, and they were excluded.

### Statistical treatment of data.

Statistical analyses were conducted using JMP Pro 15 (SAS Institute, Inc.). Concentrations of V. vulnificus (CFU or *vvhA* gene copies · mL^−1^), total bacteria, Chl *a*, nutrients, PC, PN, and %C-type data were log-transformed, and rainfall and streamflow were cube or sixth-root transformed, to normalize the data prior to linear correlation, least-squares regression, and multivariate analyses. For some analyses, sites were clustered into categories of “Upper canal” (Sites 1 and 5 to 8) and “Lower canal” (Sites 10, 11, and 13 to 15) based on whether they were landward or seaward of the sediment sill deposited at the mouth of the Mānoa-Pālolo Stream. Percent C-type data were calculated only for samples for which both *vvhA* and *vcgC* values were above the reporting limit and capped at 100%. Comparison of 24-h antecedent rainfall in nominal dry and rainy seasons used a single averaged value for each sampling event, and excluded trihoral sampling, which occurred only in the dry season. Comparison of means between two sets of samples were conducted with Welch’s *t* tests to accommodate unequal variance. Comparisons of means among three or more samples were conducted by ANOVA with a *post hoc* Tukey-Kramer test of honestly significant difference. Factor analysis was conducted on *vvhA* and nutrient data using principal components with varimax rotation. For multiple linear regression, the data were split into two subsets (salinity of <12 or ≥12 ppt) because of the nonlinearity in the relationship between V. vulnificus and salinity (22). Multiple linear regression models were also generated for data covering the entire salinity range by including either a quadratic term for salinity (26, 58) or a derived variable, ΔSal_opt_, which is the absolute value of difference between the sample salinity and an optimum salinity set as 12 ppt (47). Variables for constructing generalized regression models on each subset were selected using the Akaike Information Criterion by screening for the subsets that produced the best fit among all possible models. Among equivalent subsets in the “green zone” (AICc to AICc + 4), either the subset with the best fit or the one with the fewest variables was selected, as noted in the text.

### Data availability.

A comma-separated values file of the data set is available at https://datadryad.org (https://doi.org/10.5061/dryad.0k6djhb1v).
