# Supplementary material for: Variable Freshwater Influences on the Abundance of Vibrio vulnificus in a Tropical Urban Estuary
Source: Appl Environ Microbiol. 2022 Mar 22;88(6):e01884-21. doi: 10.1128/aem.01884-21 (PMC8939318; doi:10.1128/aem.01884-21)
Supplement: Supplemental fIle 1 — Tables S1 to S5 and Fig. S1 to S6. Download AEM.01884-21-s0001.pdf, PDF file, 1.2 MB [file aem.01884-21-s0001.pdf]

Supplemental Tables and Figures

Table S1. Ala Wai Canal sampling sites coordinates and descriptions

Site	Latitude	Longitude	Location Description†
1	21.275249	-157.818151	Upper, terminal end, near storm drains
5	21.275510	-157.817869	Upper, north side
6	21.275000	-157.817452	Upper, terminal end, near storm drains
7	21.275492	-157.818411	Upper, south side
8	21.276119	-157.818461	Upper, north side, near golf course drainage culvert
9	21.283173	-157.824509	Mānoa-Palolo Stream mouth
10	21.282923	-157.826962	Lower, south side
11	21.288152	-157.832155	Lower, north side, near McCully St. Bridge
12	21.289417	-157.834312	Makiki Stream mouth
13	21.289058	-157.834602	Lower, north side, near Kalakaua Ave. Bridge
14	21.287766	-157.839309	Lower, south side, near Ala Moana Blvd Bridge
15	21.283681	-157.839639	Lower, south side inner Ala Wai Harbor

† “Upper” refers to stations at the upper terminal end of the canal, up-canal from the Mānoa-Palolo Stream inlet and the sediment sill. “Lower” refers to canal stations seaward of the Mānoa-Palolo stream. “North side” and “south side” refer to the shore of the canal from which samples were collected.

Table S2. Pairwise correlations among cube-root transformed 24-hr antecedent rainfall (Mānoa Valley), sixth-root transformed 24-hr average streamflow (Mānoa-Palolo Stream) on the day of sampling, and canal-wide averaged salinity and temperature for each of the thirteen monthly samplings. All correlations were significant ($p \leq .002$) and shown in black for positive, red for negative relationships.

	$\sqrt[3]{Rainfall}$	$\sqrt[6]{Streamflow}$	**Salinity**
$\sqrt[6]{Streamflow}$	**0.87**		
**Salinity**	**-0.85**	**-0.89**	
**Temperature**	**-0.83**	**-0.77**	**0.81**

Table S3. Pairwise correlations (lower left of the matrix) and partial correlations (upper right of the matrix) among variables. Correlations that are significant ($p \leq .01$) are in bold (black for positive, red for negative) and those that are not significant are in grey.

	Log(*vvhA*)	Temp	Salinity	Log(NO_3^-)	Log(NO_2^-)	Log(NH_4^+)	Log(PO_4^{-3})	Log(Si)	Log(PC)	Log(Chl*a*)	Log(bact)
Log(*vvhA*)		**0.25**	**-0.53**	-0.082	**0.25**	**0.18**	**0.33**	-0.021	**-0.40**	**0.24**	**0.30**
Temp	**-0.17**		**0.42**	**-0.25**	**-0.20**	0.035	-0.041	**0.24**	0.154	0.022	**0.13**
Salinity	**-0.51**	**0.65**		-0.12	**0.27**	-0.060	0.159	**-0.43**	**-0.77**	**0.70**	**0.34**
Log(NO_3^-)	**0.33**	**-0.29**	**-0.32**		**0.26**	0.017	**0.23**	**0.23**	-0.049	**0.20**	0.14
Log(NO_2^-)	**0.45**	-0.14	-0.16	**0.51**		**0.19**	0.011	**0.31**	0.14	-0.17	0.098
Log(NH_4^+)	**0.48**	**-0.23**	**-0.44**	**0.31**	**0.39**		0.025	0.091	0.098	-0.061	-0.11
Log(PO_4^{-3})	**0.41**	**-0.29**	**-0.46**	**0.40**	**0.25**	**0.34**		0.082	**0.38**	**-0.20**	**-0.33**
Log(Si)	**0.47**	0.007	-0.14	**0.43**	**0.53**	**0.30**	0.11		**-0.57**	**0.41**	**0.19**
Log(PC)	-0.061	-0.001	**-0.25**	0.18	-0.042	0.086	**0.33**	**-0.32**		**0.79**	**0.47**
Log(Chl*a*)	**-0.25**	**0.50**	**0.49**	0.086	-0.039	-0.18	-0.072	-0.071	**0.54**		**-0.18**
Log(bact)	0.020	**0.41**	**0.29**	0.13	0.17	-0.076	-0.13	0.066	**0.37**	**0.51**	

Table S4. Oligonucleotide primer and probe sequences used in Taqman real-time qPCR amplification of *vvhA* gene for total *V. vulnificus* and *vcgC* gene for C--Type *V. vulnificus*.

	vvhA (total *V. vulnificus*)[1]	***vcgC*** (Type-C *V. vulnificus*)[2]
Primer (F)	TGT TTA TGG TGA GAA CGG TGA	AAA ACT CAT TGA RCA GTA ACG AAA
Reverse (R)	TTC TTT ATC TAG GCC CCA AAC TTG	GCT GGA TCT AAK CCC AAT GC
Probe	*FAM*–CCG TTA ACC GAA CCA CCC GCA A–*TAMRA*	*FAM*–AAT TAA AGC CGT CAA GCC ACT GAC TGT AAA GAA–*TAMRA*

[1] Campbell MS, Wright AC. 2003. Real-time PCR analysis of Vibrio vulnificus from oysters. Appl Environ Microbiol 69:7137–7144.

[2] Baker-Austin C, Gore A, Oliver JD, Rangdale R, McArthur JV, Lees DN. 2010. Rapid in situ detection of virulent Vibrio vulnificus strains in raw oyster matrices using real-time PCR. Env Microbiol Rep 2:76–80.

Table S5. Efficiency and intercepts of the standard curves for all qPCR assays from which data were derived for this study.

***vvhA* Assay Run**	**Efficiency**	**Intercept**	***vcgC* Assay Run**	**Efficiency**	**Intercept**
P3_5_9_10	97%	40.3	P1_5_6_7	101%	42.3
P2_1_2_3	103%	40.2	P1_8_9_10	108%	42.2
P2_5_9_10	99%	40.3	P3_7_8_P1_1	110%	41.7
P2_4_6_7	104%	39.8	P2_8_P3_1_2	110%	42.1
P2_8_9R_P3_1_2	102%	40.0	P3_3_4_6	106%	42.2
P3_3_4_6	99%	40.3	P2_1_2_3	107%	42.5
P3_7_8_P1_1	104%	39.5	P2_4_6_7	109%	42.4
P1_2_3_4	98%	40.2	P2_5_9_10	100%	42.7
P1_8_9_10	103%	40.0	P1_2_3_4	101%	42.6
Redo1	101%	40.6	P3_5_9_10	106%	42.3
Redo2	104%	39.8	Redo1	106%	42.4
Average	101%	40.1	Average	106%	42.3
Standard Deviation	3%	0.32	Standard Deviation	4%	0.27
Maximum	104%	40.6	Maximum	110%	42.7
Minimum	97%	39.5	Minimum	100%	41.7

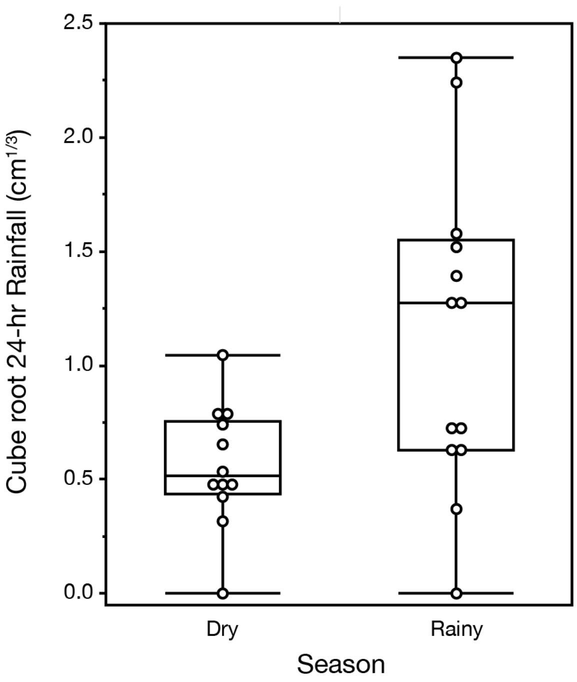

Fig. S1. Rainfall in rainy vs. dry seasons. Cube-root transformed data points represent the average 24-hour antecedent rainfall during each sampling event except trihoral samplings which were conducted only in the dry season. Points are shown (jittered to avoid overlap) along with box plots illustrating range, 25%, 75% quantiles and median . Rainfall was significantly higher on average for months in the nominal dry (Apr–Sep; n = 12) vs. rainy (Oct–Mar; n = 13) seasons (Welch’s t-test for samples with unequal variances, $p = .014$).

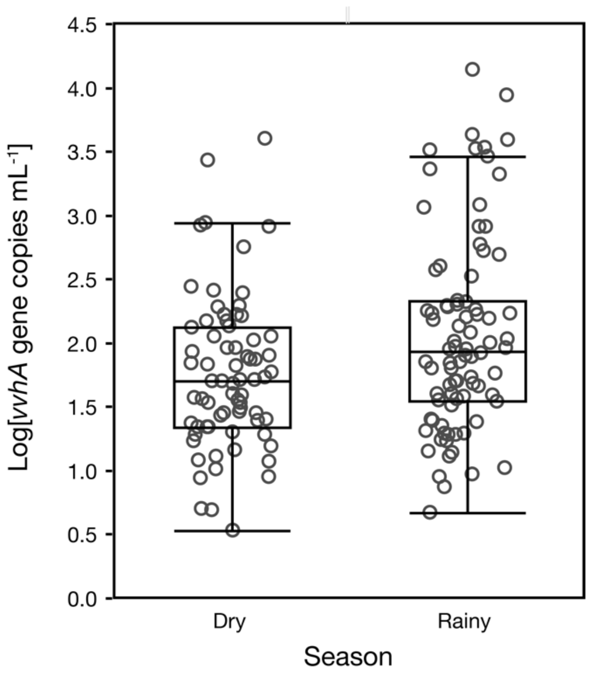

Fig. S2. Log-transformed *vvhA* gene copies mL^{-1} for monthly samplings in rainy vs. dry seasons. Points are shown (jittered to avoid overlap) along with box plots illustrating range, 25%, 75% quantiles and median . The average concentration of *vvhA* gene was significantly higher for months in the nominal rainy (Oct–Mar; n = 84) vs. dry (Apr–Sep; n = 71) seasons (Welch’s t-test for samples with unequal variances, p = .0065)

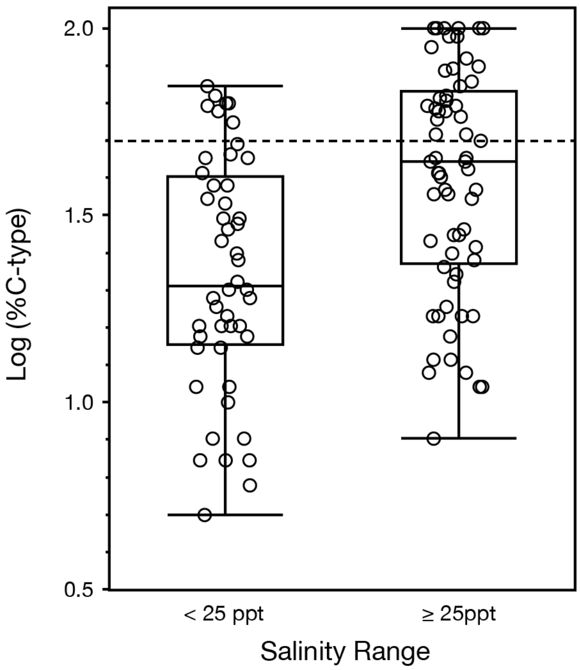

.

Fig. S3. Percentage of detected *V. vulnificus* that are C-type at nominal low (<25 ppt; n = 48) vs. high (≥ 25 ppt; n = 65) salinity. Log-transformed % C-type data is shown for all cases where both *vvhA* and *vcgC* data were above reporting limits. Points are shown (jittered to avoid overlap) along with box plots illustrating range, 25%, 75% quantiles and median. The dotted reference line indicates 50% C-type. The estimated %C-type was significantly higher on average in high salinity samples (Welch’s t-test, $p < .0001$).

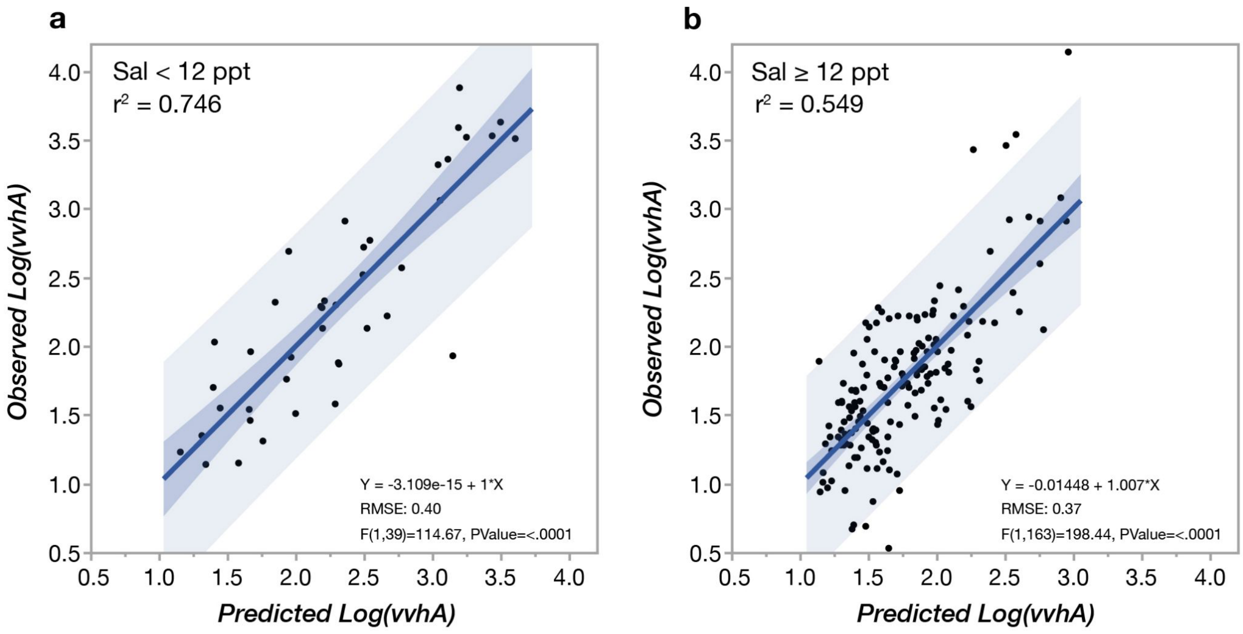

Fig. S4. Observed vs. predicted log(*vvhA*) for individual samples using **a**) a model for samples with salinity < 12 ppt and **b**) a model for samples with salinity ≥ 12 ppt. The equation for the predicted values in **a** is: $log[vvhA] = 0.154 \bullet T + 1.015 \bullet log[nitrite] - 0.600 \bullet log[silica] - 0.850 \bullet log[PC] + 2.170$ where T is temperature in °C, and nitrite, silica, and particulate carbon (PC) are in units of µM. The equation for predicted values in **b** is: $log[vvhA] = 0.0360 \bullet T - 0.0727 \bullet S + 0.515 \bullet log[phosphate] + 2.835$ where T is temperature in °C, S is salinity is in units of ppt, and phosphate is in units of µM. Combining predictions from both models resulted in an overall relationship with r2 = 0.667 (Fig. 5, main text).

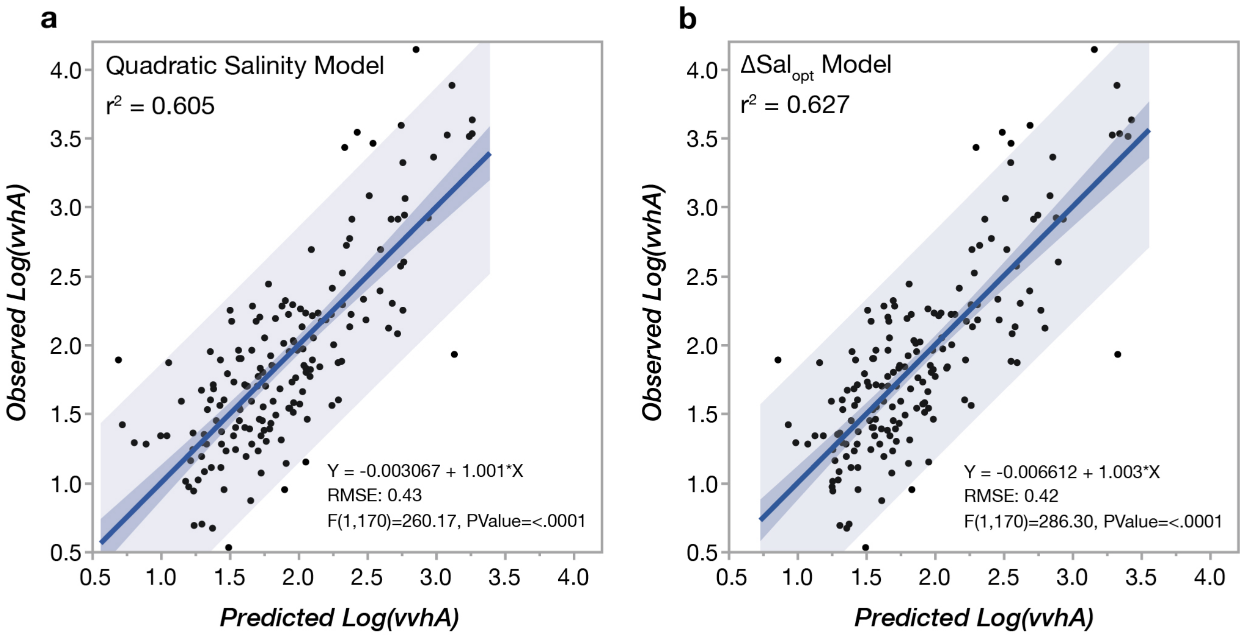

Fig. S5. Observed vs. predicted log(*vvhA*) for individual samples using **a**) a model that includes a quadratic term for salinity or **b**) a term for distance from optimal salinity, $\mathbf{\Delta Sal_{opt}}$. The equation for data shown in **a** is: $log[vvhA] = 0.0835 \bullet T - 0.0406 \bullet S - 0.002 \bullet S^2 + 0.599 \bullet log(nitrite) + 0.500 \bullet log(phosphate) - 0.372 \bullet log(PC) + 1.122$ where T is temperature in °C, S salinity in ppt, and nitrite, phosphate and PC are in μM. The equation for predictions in **b** is: $log[vvhA] = 0.067 \bullet T - 0.071 \bullet \Delta Sal_{opt} + 0.501 \bullet log(nitrite) + 0.48 \bullet log(phosphate) - 0.268 \bullet log(PC) + 1.819$ where T is temperature in °C, ΔSal_{opt} is in ppt, and nitrite, phosphate and PC are in μM.

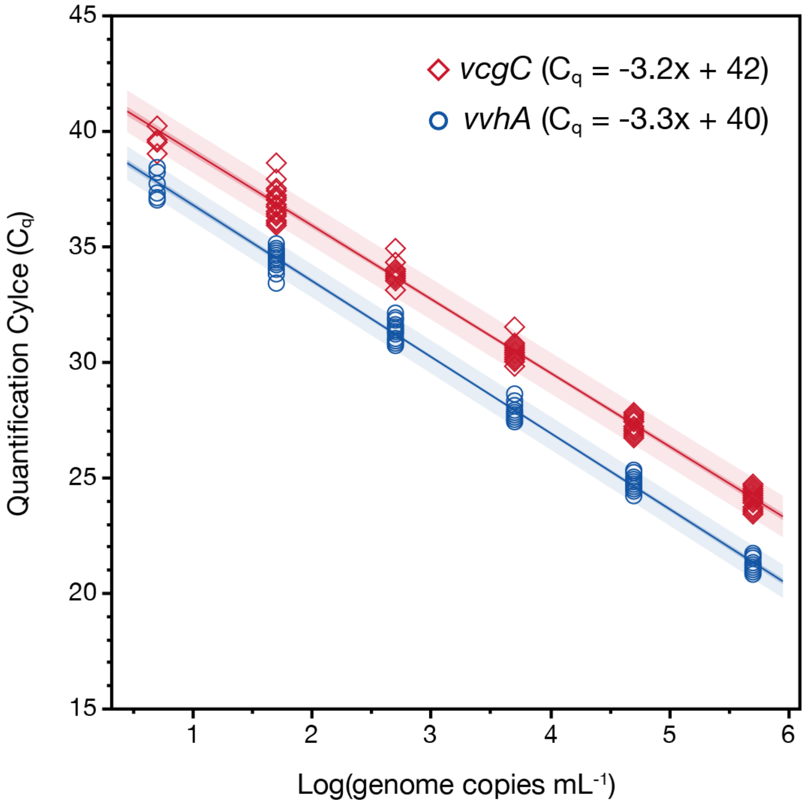

Fig. S6. Compiled data for all standard curves for *vvhA* (blue) and *vcgC* (red) genes. Standard curve points were assayed in duplicate for eleven different assay runs per gene and all individual standard curve points are shown along with an overall linear regression fit for all data for each gene. Shaded areas indicate the 95% confidence limits for the fit of the line (darker shading very close to each line) and predicted value (lighter shading). Slopes and intercepts for the curves for each independent run are summarized in Supplementary Table S3.

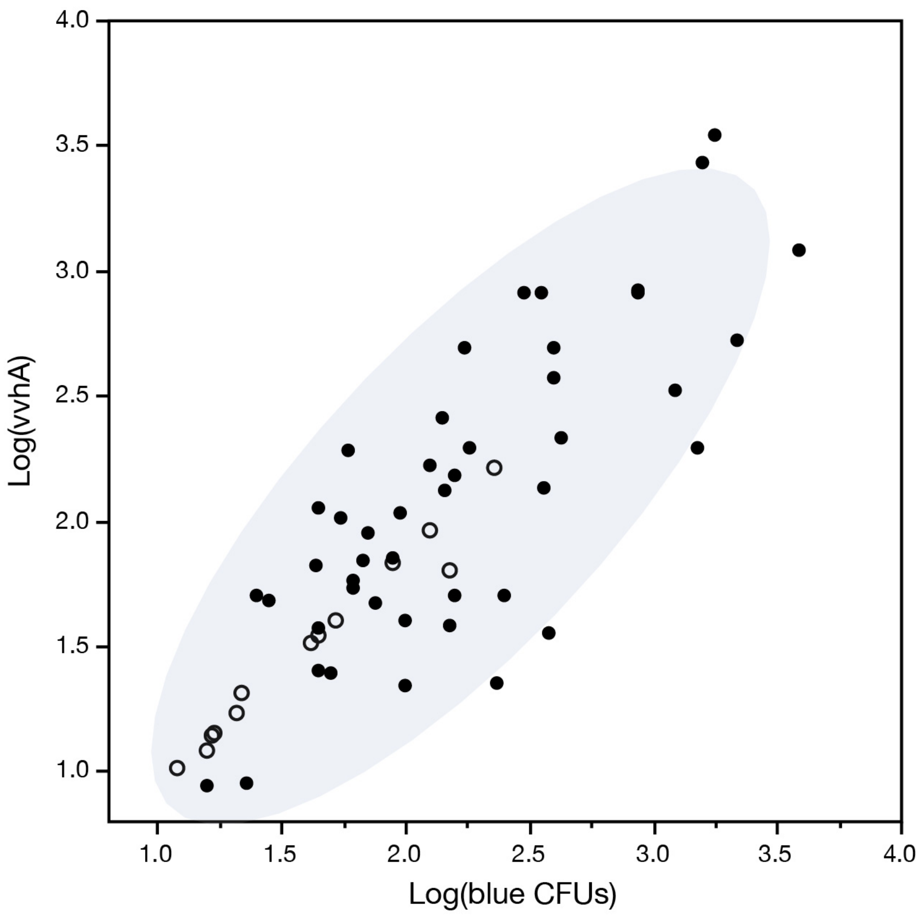

Fig. S7. Correlation between concentrations of blue colony-forming units on CRHOMagar™ Vibrio medium and qPCR estimates of *vvhA* gene copies ($r = 0.79$, $n = 58$, $p < .0001$). Correlation was calculated only for the solid symbols for which both qPCR and CFU data were available. Open symbols illustrate cases where qPCR data were unavailable or low confidence, so CFU data were used alone or in combination with qPCR data to estimate *vvhA*.
